# Scar Sarcoidosis at the Site of Liposuction With Progression to Systemic Sarcoidosis

**DOI:** 10.7759/cureus.78844

**Published:** 2025-02-11

**Authors:** Ashley N Houff, Sumalatha Nandikonda, Neha Bhanusali, Maria Farooq, Shazia Beg

**Affiliations:** 1 Dermatology, University of Central Florida College of Medicine, Orlando, USA; 2 Rheumatology, University of Central Florida College of Medicine, Orlando, USA

**Keywords:** dermatology, monitoring, rheumatology, scar, scar sarcoidosis, systemic sarcoidosis

## Abstract

Sarcoidosis is a multisystem autoimmune disease characterized by the formation of granulomas that can either be limited to the skin as cutaneous sarcoidosis or have systemic involvement affecting multiple organs. Here, we present the case of a 46-year-old female patient who presented with erythematous, non-tender, enlarging skin nodules at the site of a previous liposuction surgery. A biopsy of the skin nodule suggested cutaneous sarcoidosis. Years later, she then developed symptoms of abdominal pain, joint pain, and dyspnea. Further workup revealed progression to systemic sarcoidosis involving multiple organs. Our report emphasizes the importance of including sarcoidosis in the differential for new, enlarging nodules at the sites of previous surgeries. Suspicious skin lesions should be biopsied promptly to help ensure early detection and treatment. We also discuss the importance of continued monitoring of patients with cutaneous sarcoidosis for signs of progression to systemic involvement.

## Introduction

Sarcoidosis is a systemic autoimmune disease characterized by the formation of granulomas affecting multiple organ systems within the body [1]. The most common sarcoidosis location, affecting approximately 90% of patients, is the lung parenchyma or hilar lymph nodes [1]. Some other most common sites of involvement are the skin and eyes [1]. However, sarcoidosis granulomas have been reported in essentially all organs previously including the heart, muscles, central nervous system, spleen, and liver [1]. Any lymph node may also be affected [1].

Diagnosis of sarcoidosis is often made clinically in combination with biopsy results showing non-caseating granulomas ruling out other diseases and imaging findings supportive of the diagnosis [2]. A sarcoidosis diagnosis score (SDS) can be used to help differentiate sarcoidosis from other granuloma-forming etiologies, such as tuberculosis [3]. However, while helpful for differentiation from infectious granulomatous diseases like tuberculosis, SDS may not be enough to differentiate from noninfectious granuloma-forming diseases such as berylliosis [4]. Therefore, environmental exposures should still be considered [4].

The prevalence of sarcoidosis in the United States is estimated to be approximately 60 per 100,000 persons [5]. The prevalence is highest in African American women, estimated to be around 178.5 per 100,000 persons [5]. Estimates of cutaneous involvement vary, but the prevalence is estimated to be around 20-25% [1,5].

Cutaneous sarcoidosis lesions have a wide range of morphologies. The most common specific cutaneous presentations are maculopapular and nodular, while a common non-specific presentation is erythema nodosum [6]. Rarer presentations are plaques, lupus pernio, and within scars [6]. Scar sarcoidosis can occur at sites of previous trauma to the skin, sites of previous surgeries, in tattoos, or at sites of foreign bodies [7]. Scar sarcoidosis is reported rarely in the literature, with varying types of surgeries prior to presentation including skin grafting and eyelid surgery [8,9].

Here, we present a rare case of scar sarcoidosis presenting after liposuction. The patient did not follow up and presented again years later with systemic sarcoidosis symptoms and involvement. We highlight the importance of early detection of scar sarcoidosis and close monitoring for systemic symptoms and involvement.

## Case presentation

**Figure 1:**
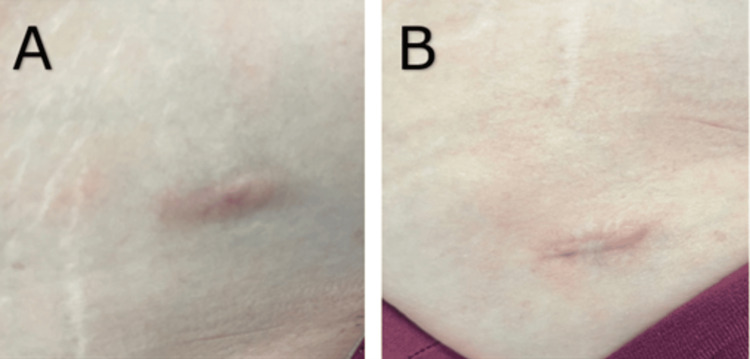
Surgical scar with nodules of the right groin (A) and right lower quadrant (B)

A 46-year-old female patient with no significant past medical history was referred to our rheumatology clinic for systemic sarcoidosis. Nineteen years prior to presenting to the clinic, she had liposuction in the bilateral lower abdominal quadrants and bilateral lower back at the dimples of Venus with remnant surgical scars. Ten years after the liposuction surgery, she developed erythematous, non-tender, enlarging skin nodules along the surgical sites. The physical exam was remarkable for pinkish nodules noted on the previous scars in bilateral lower quadrants, bilateral thighs, and bilateral dimples of Venus (Figure 1). Biopsy of the nodules showed non-caseating granulomas, which was suspicious for sarcoidosis.

**Figure 2:**
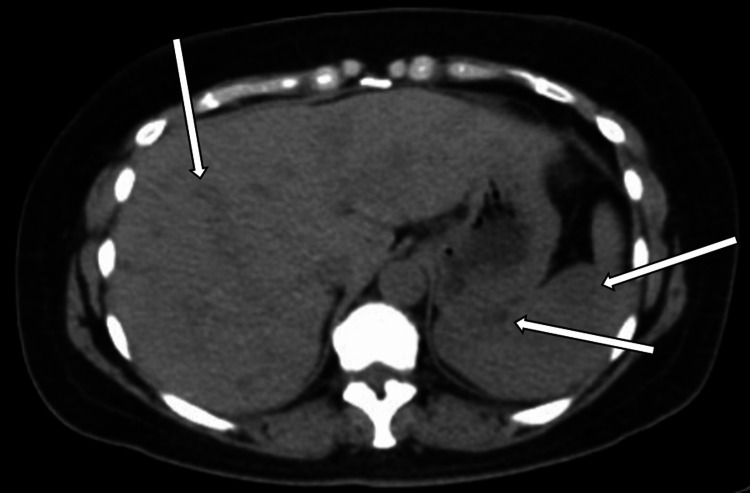
Abdominal CT scan showing multiple nodules of the liver and spleen (white arrows) CT: computed tomography

Despite suspicion for cutaneous sarcoidosis, the patient did not follow up until seven years later when she started to develop signs of systemic involvement. She developed right upper quadrant pain, dyspnea, and joint pain in her knees, back, and hips. She also noticed new nodules at other liposuction sites. At this time, an abdominal computed tomography (CT) scan showed multiple liver and spleen nodules (Figure 2). CT scan also showed small nodules of the lungs, though these nodules were less specific.

**Figure 3:**
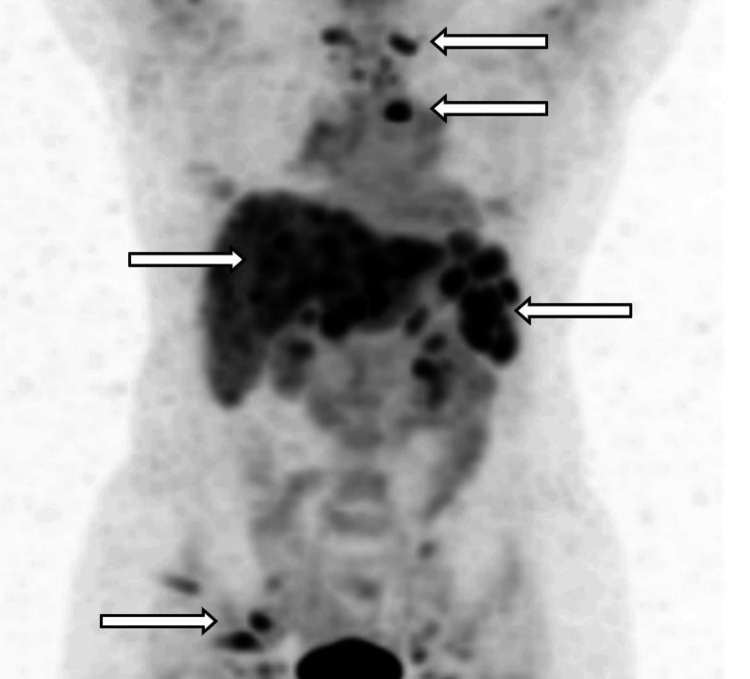
PET scan showing hypermetabolic adenopathy of the neck, chest, abdomen, and pelvis as well as multiple hypermetabolic lesions in the liver and spleen (white arrows) PET: positron emission tomography

Positron emission tomography (PET) scan revealed hypermetabolic adenopathy within the neck, chest, abdomen, and pelvis as well as multiple hypermetabolic lesions in the liver and spleen with primary consideration for metastatic disease, lymphoma, and sarcoidosis (Figure 3).

**Figure 4:**
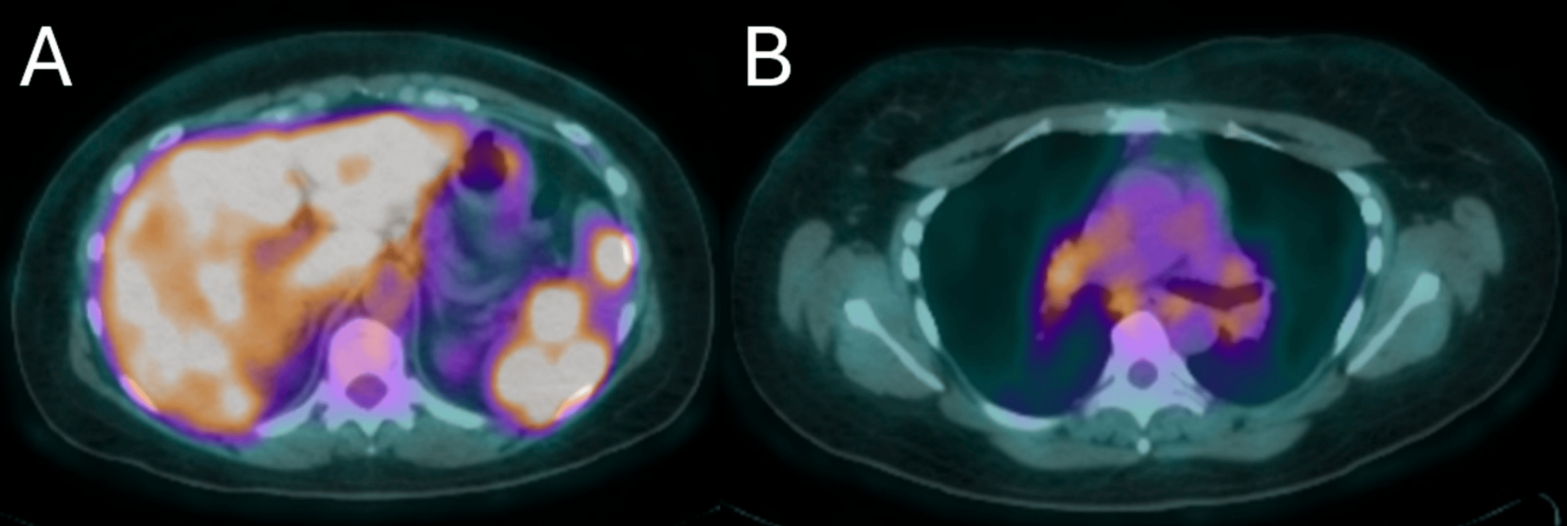
Colorized PET scan images showing hypermetabolic areas of the liver, spleen, and chest PET: positron emission tomography

Increased metabolic activity can also be seen in the colorized PET scan images (Figure 4).

**Figure 5:**
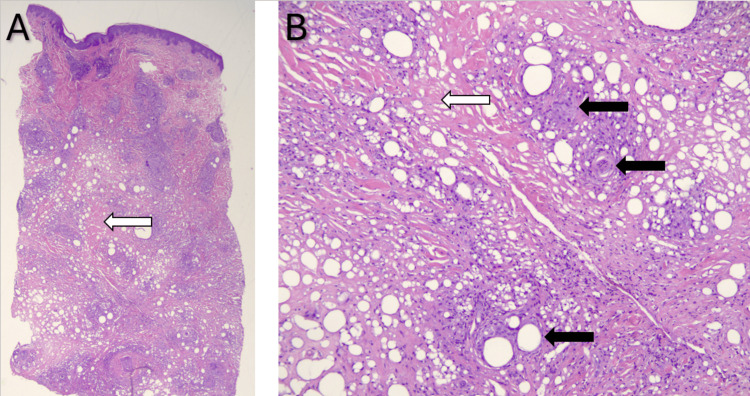
Skin nodule biopsy H&E staining demonstrating fibrosis (white arrows) and non-caseating granulomas (black arrows) at 1× (A) and 10× (B) magnification H&E: hematoxylin and eosin

Liver biopsy showed several complex non-caseating granulomas, suggestive of systemic sarcoidosis. Repeat skin biopsy of the right lower quadrant nodule showed sarcoid-like granulomas involving the full thickness of the dermis associated with a low number of non-atypical lymphocytes (Figure 5). Laboratory workup revealed elevated angiotensin-converting enzyme (ACE) level (110 nmol/mL/min, normal <40). The patient did not have anemia, leukopenia, or thrombocytopenia. 

**Figure 6:**
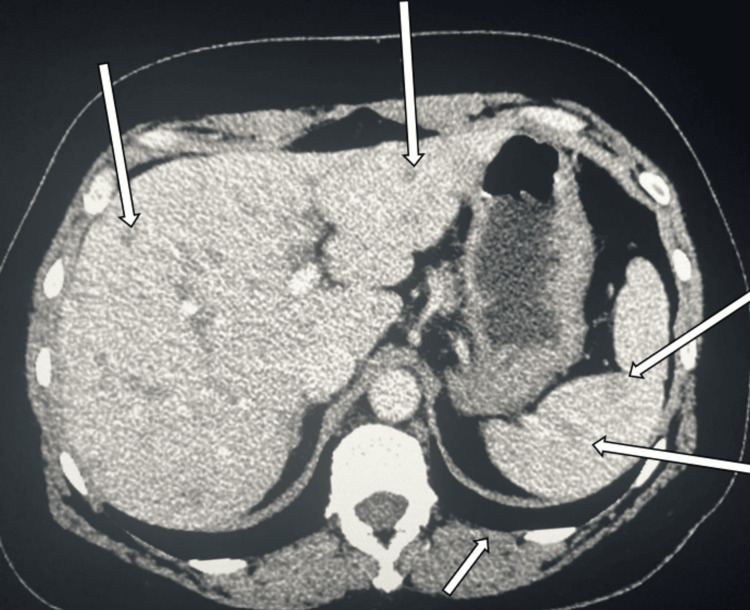
Repeat abdominal CT scan showing stable nodules of the liver and spleen (white arrows) CT: computed tomography

The patient was diagnosed with systemic sarcoidosis and was started on prednisone 30 mg daily for one month with a taper of 20 mg daily for one month, 10 mg daily for one month, 5 mg daily for one month, and then discontinued. The prednisone improved her symptoms of shortness of breath and joint pain. The sarcoid nodules at the scar sites decreased in size. Repeat ACE level was normal. One year later, a repeat abdominal CT scan showed stable lymphadenopathy and nodules (Figure 6).

Due to the systemic involvement of the sarcoidosis, the patient was started on methotrexate 15 mg by mouth once weekly. She did not tolerate oral methotrexate, so she was switched to the injectable form, methotrexate 20 mg subcutaneous injection once weekly and folic acid daily, with better tolerability. Due to the recurrence of joint pains, she was also restarted on prednisone 5 mg daily for one month and then as needed for joint pain. Based on her response to methotrexate as measured symptomatically and with imaging, she may be started on a tumor necrosis factor (TNF)-alpha inhibitor.

## Discussion

Although the majority of cases of skin sarcoidosis tend to be mild and may not need treatment, skin eruptions, as seen in our patient, may be associated with the involvement of other organ systems and may be one of the first signs of systemic sarcoidosis, warranting further workup [6]. One prospective study of patients who had systemic sarcoidosis with cutaneous involvement found that 26% of the patients had their cutaneous symptoms present prior to the diagnosis of systemic sarcoidosis [10]. Many (74%) had cutaneous symptoms present at the time of diagnosis of systemic involvement, while 20% developed cutaneous lesions after their systemic sarcoidosis diagnosis [10]. For patients presenting initially with cutaneous signs, the average time to diagnosis of systemic sarcoidosis was 22 months [10]. However, as our case demonstrated with systemic symptoms presenting seven years after cutaneous symptoms, systemic signs can occur years after cutaneous involvement.

Our patient's delay in follow-up after cutaneous sarcoidosis treatment led to systemic involvement only being detected after systemic symptoms started. It is recommended to perform screening for systemic involvement initially at cutaneous presentation, including but not limited to chest radiograph, electrocardiogram, echocardiogram, pulmonary function testing, urinalysis, and ophthalmologic examination [11]. As in our case, imaging can detect the involvement of sarcoidosis systemically as well as monitor for improvement with treatment. It is important for the clinician diagnosing cutaneous sarcoidosis to emphasize the importance of screening for systemic involvement to avoid delay in detection and treatment.

## Conclusions

Our report adds to the growing body of reports of scar sarcoidosis progressing to systemic involvement. This clinical case of cutaneous sarcoidosis highlights the importance of having suspicion for sarcoidosis when a patient has recurrent nodules in an area of a surgical scar. It is important to keep cutaneous sarcoidosis as a differential diagnosis to reduce the delay in diagnosis. Any patient with cutaneous sarcoidosis needs an initial workup for systemic involvement, followed by periodic screening as systemic involvement can occur years after initial cutaneous involvement.

## References

[1] Husain AN (2020). The lung: granulomatous diseases. Robbins & Cotran Pathologic Basis of Disease.

[2] (1999). Statement on sarcoidosis. Joint Statement of the American Thoracic Society (ATS), the European Respiratory Society (ERS) and the World Association of Sarcoidosis and Other Granulomatous Disorders (WASOG) adopted by the ATS Board of Directors and by the ERS Executive Committee, February 1999. Am J Respir Crit Care Med.

[3] Bickett AN, Lower EE, Baughman RP (2018). Sarcoidosis diagnostic score: a systematic evaluation to enhance the diagnosis of sarcoidosis. Chest.

[4] Jeny F, Vucinic V, Zhou Y (2023). Validation of the sarcoidosis diagnostic score in a multicontinental study. Ann Am Thorac Soc.

[5] Baughman RP, Field S, Costabel U (2016). Sarcoidosis in America. Analysis based on health care use. Ann Am Thorac Soc.

[6] Lodha S, Sanchez M, Prystowsky S (2009). Sarcoidosis of the skin: a review for the pulmonologist. Chest.

[7] Imadojemu S, Wanat KA, Noe M, English III JC, Rosenbach M (2019). Cutaneous sarcoidosis. Sarcoidosis: A Clinician's Guide.

[8] Xiao A, Falcone LM, English Iii JC (2023). Systemic sarcoidosis presenting in a scar. Case Rep Dermatol Med.

[9] Suh SY, Ahn JH (2021). Scar sarcoidosis with systemic involvement after blepharoplasty. Int J Ophthalmol.

[10] Marcoval J, Mañá J, Rubio M (2011). Specific cutaneous lesions in patients with systemic sarcoidosis: relationship to severity and chronicity of disease. Clin Exp Dermatol.

[11] Haimovic A, Sanchez M, Judson MA, Prystowsky S (2012). Sarcoidosis: a comprehensive review and update for the dermatologist: part II. Extracutaneous disease. J Am Acad Dermatol.

